# The autophagy-independent role of BECN1 in colorectal cancer metastasis through regulating STAT3 signaling pathway activation

**DOI:** 10.1038/s41419-020-2467-3

**Published:** 2020-05-01

**Authors:** Fuqing Hu, Geng Li, Changsheng Huang, Zhenlin Hou, Xi Yang, Xuelai Luo, Yongdong Feng, Guihua Wang, Junbo Hu, Zhixin Cao

**Affiliations:** Department of Gastrointestinal Surgery Center, Tongji Hospital, Tongji Medical College, Huazhong University of Science and Technology, Wuhan, Hubei 430030 China

**Keywords:** Colon cancer, Cell invasion

## Abstract

BECN1 is a critical regulator of autophagy, which plays important roles in tumor formation and metastasis. However, the autophagy-independent role of BECN1 and the clinical prediction value of BECN1 still need to be explored. Here, we observed significantly lower expression of BECN1 in colorectal cancers (CRCs) compared with adjacent normal colon tissue, and downregulation of BECN1 was positively related to poor prognosis in CRC patients. In addition, we found that knockdown of BECN1 markedly promoted CRC cell motility and invasion. Bioinformatics gene set enrichment analysis (GSEA) revealed that low levels of BECN1 were significantly correlated with the STAT3 signaling pathway in CRC. Consistently, knockdown of BECN1 increased the phosphorylation of STAT3 and activated the STAT3 signaling pathway in CRC cells. Furthermore, we demonstrated that STAT3 was involved in the CRC metastasis mediated by knockdown of BECN1 in vitro and in vivo. Mechanistically, knockdown of BECN1 promoted the phosphorylation of STAT3 via regulation of the interaction between STAT and JAK2 but did not inhibit autophagy. Our study revealed that BECN1 served as a negative regulator of CRC metastasis by regulating STAT3 signaling pathway activation in an autophagy-independent manner. The BECN1/JAK2/STAT3 signaling pathway can be used as a potential therapeutic target for metastatic CRC.

## Introduction

BECN1, which contains a Bcl-2 homology (BH3) domain, a coiled-coil domain (CCD) and an evolutionarily conserved domain (ECD), has been identified as an important autophagy effector and plays a critical role in autophagy^1^. BECN1 interacts with Vps34 and other factors to regulate autophagosome formation^2–4^. In addition, BECN1 was also reported to bind to Bcl family proteins, such as Bcl-2 and Bcl-XL, which in turn counteracted the effect of BECN1 on autophagy^5–7^. Autophagy is a survival-promoting pathway that captures, degrades, and recycles intracellular proteins and organelles in lysosomes^8–10^. Upregulated autophagy in cancers facilitates cancer cell survival upon microenvironmental stress and increases cancer growth and aggressiveness^11,12^. As a key factor in autophagy, BECN1 should act as an oncogene in cancer, but the roles of BECN1 in cancers are still unclear. In breast cancer, loss of BECN1 promotes mammary tumor development via regulation of WNT1^13^, while it has also been demonstrated that BECN1 is essential for the tumorigenesis of breast cancer progenitor cells^14^. Previous reports have also shown that heterozygous disruption of the BECN1 autophagy gene increases spontaneous tumorigenesis^15^. In gastric cancer, low expression of BECN1 was associated with a malignant phenotype and poor prognosis^16^. High expression of Beclin-1 predicted a good overall survival rate in non-Hodgkin’s lymphomas^17^, suggesting that BECN1 is a tumor suppressor. These studies also suggest that BECN1 might have some autophagy-independent functions, which would imply a different role for BECN1 in cancer; these ideas need to be explored.

Signaling transducer and activator of transcription (STAT) proteins are major transcription factors that respond to the activation of different cytokine (IL-6 or IFN) receptors and translocate to the nucleus, where they readily activate DNA transcription^18^^,19^. STAT3, one of most studied STAT proteins, is continually activated in a wide range of tumors and involved in different steps of cancer progression, including proliferation, apoptosis, maintenance of cancer stemness, and metastasis^20,21^. Elevated phosphorylation levels of STAT3 have been associated with poor prognosis and increased metastasis in brain tumors^22^. It has also been reported that the IL-6/STAT3 pathway plays an essential role in tumor progression in CRC^23,24^.

In this study, we explored the potential role of BECN1 in colorectal cancer (CRC) progression. We found that BECN1 was downregulated in clinical CRC samples and that knockdown of BECN1 promoted CRC metastasis but had no effect on CRC proliferation. Furthermore, a bioinformatics assay showed that the protein expression of BECN1 was inversely associated with the STAT3 signaling pathway. Knockdown of BECN1 enhanced the phosphorylation of STAT3 in different CRC cells. Interestingly, we showed that BECN1 affected STAT3 phosphorylation in an autophagy-independent manner. We explored how BECN1 directly interacts with STAT3 and JAK2, and knockdown of BECN1 markedly strengthened the interaction between STAT3 and JAK2, in turn enhancing the phosphorylation of STAT3.

## Results

### BECN1 is downregulated in CRC, and downregulation of BECN1 predicts poor prognosis in CRC

**Fig. 1:**
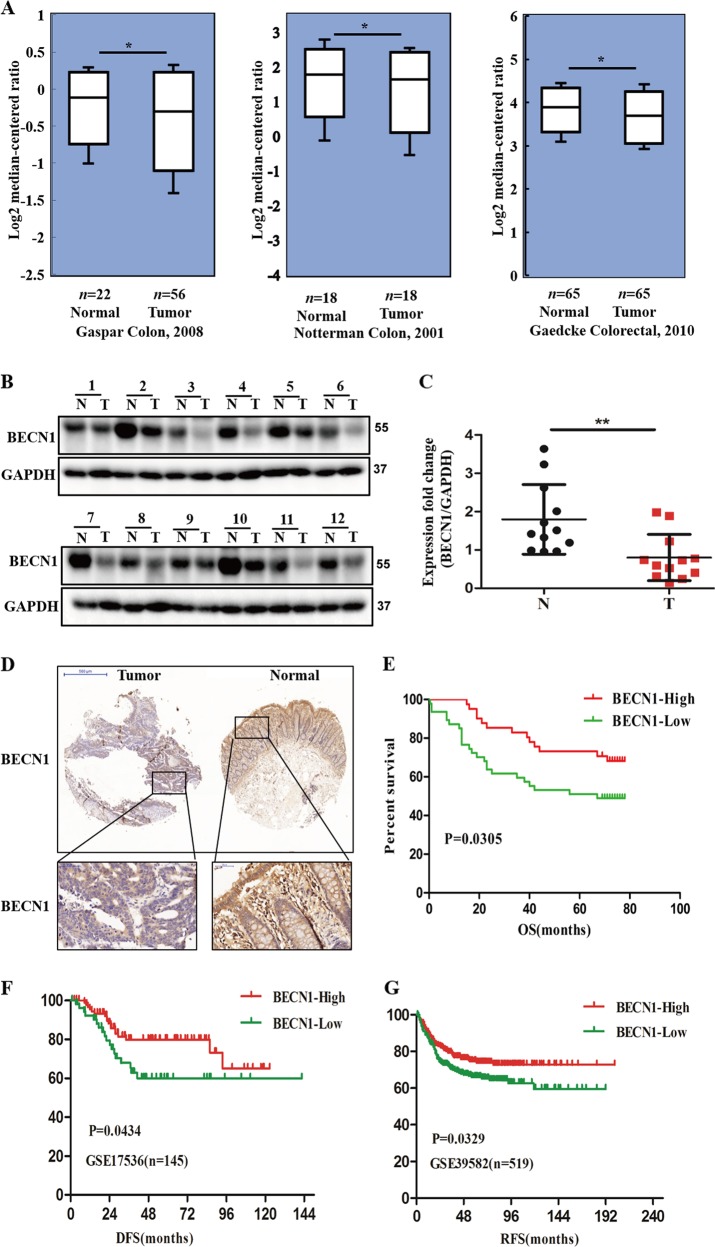
**BECN1 is downregulated in CRC, and downregulation of BECN1 predicts poor prognosis in CRC**. **a** Oncomine box plots showing that BECN1 is downregulated in different CRC datasets. **b**, **c** Western blot assay of the protein expression of BECN1 in 12 paired clinical CRC patient tissues. The densities of bands were determined by AlphaView SA software. **d** Representative IHC images of BECN1 expression in CRC tissue microarrays. **e**–**g** Kaplan–Meyer plots for survival analysis of BECN1 expression in CRC patients based on data from tissue microarrays and two online databases (GSE17536 and GSE39582).

To explore the potential role of BECN1 in CRC development, using the Oncomine expression database, we found that BECN1 was downregulated in CRC samples from various datasets (www.oncomine.org) (Fig. 1a). Consistent with the above results, the TCGA colorectal cancer RNAseq database results also indicated BECN1 downregulation in human CRC tissues (Fig. S1A). To further confirm these public database results, we examined the expression of BECN1 in human CRC tissues and showed that BECN1 was significantly downregulated in 12 CRC patient tissues compared with the paired adjacent normal tissues (Fig. 1b, c). IHC staining showed that the expression of BECN1 was markedly decreased in tumor tissues compared with the adjacent normal samples (Fig. 1b). Furthermore, we found that BECN1 had strong expression in normal tissues and weak expression in CRC specimens from the Human Protein Atlas (www.proteinatlas.org) (Fig. S1B); all of these findings indicate that BECN1 plays an important role in CRC progression.

Then, we tried to determine the prognostic value of BECN1 in CRC. We analyzed BECN1 protein levels in 134 CRC patients using tissue microarrays, and the loss of BECN1 protein expression was significantly associated with poor overall survival (Fig. 1e). By analyzing two different CRC GEO databases, we also found that lower mRNA levels of BECN1 were associated with poorer disease-free survival (DFS) and recurrence-free survival (RFS) in CRC (Fig. 1f, g). Overall, all these findings indicate that BECN1 is a potential marker for predicting clinical prognosis in CRC patients.

### Loss of BECN1 promotes cancer metastasis in CRC

**Fig. 2:**
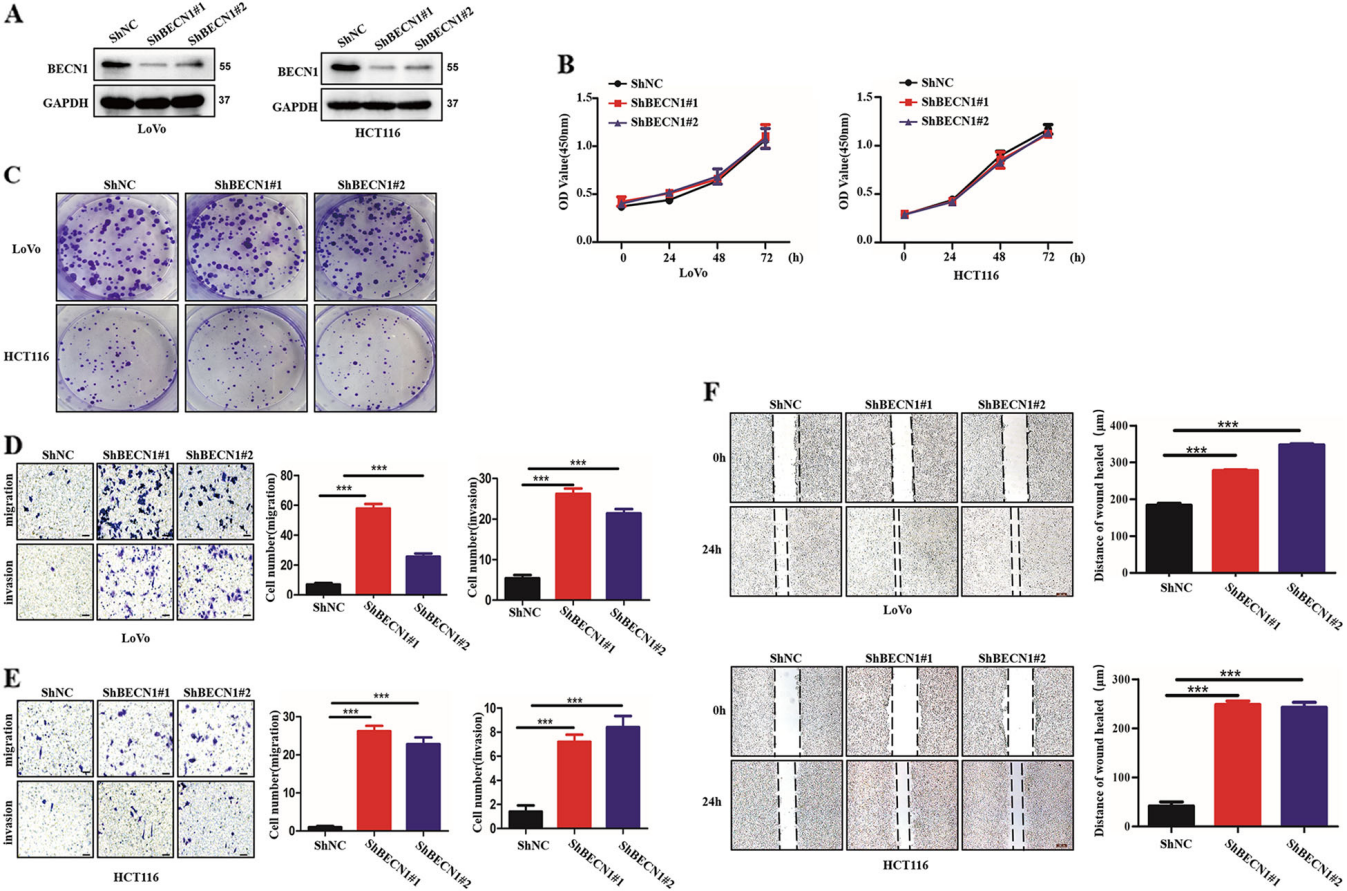
**Loss of BECN1 promotes cancer metastasis in CRC**. **a** LoVo and HCT116 cells stably expressing negative control shRNA, shRNA-BECN1#1 or shRNA-BECN1#2, as indicated, were examined by western blotting. The GAPDH protein was used as a loading control. **b** The viability of each cell line as indicated was examined by CCK8 assay in LoVo and HCT116 cells. **c** The colony-formation assay was used to evaluate cell growth in LoVo and HCT116 cells. **d**, **e** Knockdown of BECN1 significantly enhanced migration and invasion abilities, as determined by transwell assays in vitro, and the right panel shows the histograms of the results. Scale bar: 100 μm. **f** Wound-healing assays were performed in both HCT116 and LoVo cells. The right panels show the histograms of the results. The bars indicate the SD. *p < 0.05, **p < 0.01 using Student’s *t*-test. Scale bar: 200 μm.

To further determine whether BECN1 is involved in CRC progression, we first established CRC cell lines stably expressing shRNA-BECN1 to knock down the endogenous expression of BECN1 using two pairs of small hairpin RNAs (Fig. 2a). We performed the CCK8 assay and colony-formation experiments to evaluate the effect of BECN1 on the growth of CRC cells. We showed that knockdown of BECN1 had a slight influence on the rate of proliferation (Fig. 2b). Consistently, the colony formation results also demonstrated that BECN1 played a small role in the proliferation of proliferation of CRC cells (Fig. 2c). Meanwhile, we further examined the changes in the cell cycle and apoptosis among different groups, and the loss of BECN1 had no effect on the cell cycle or apoptosis (Fig. S2A, B). Interestingly, we found that LoVo and HCT116 cells expressing sh-BECN1 had an augmented invasive ability in migration and invasion assays compared with the control cells (Fig. 2d, e). The wound-healing assay also demonstrated that knockdown of BECN1 in CRC cells promoted cell migration (Fig. 2f). Taken together, these data indicate that BECN1 plays a vital role in metastasis and slightly affects cell proliferation, the cell cycle and apoptosis.

### Loss of BECN1 activates the phosphorylation of STAT3

**Fig. 3:**
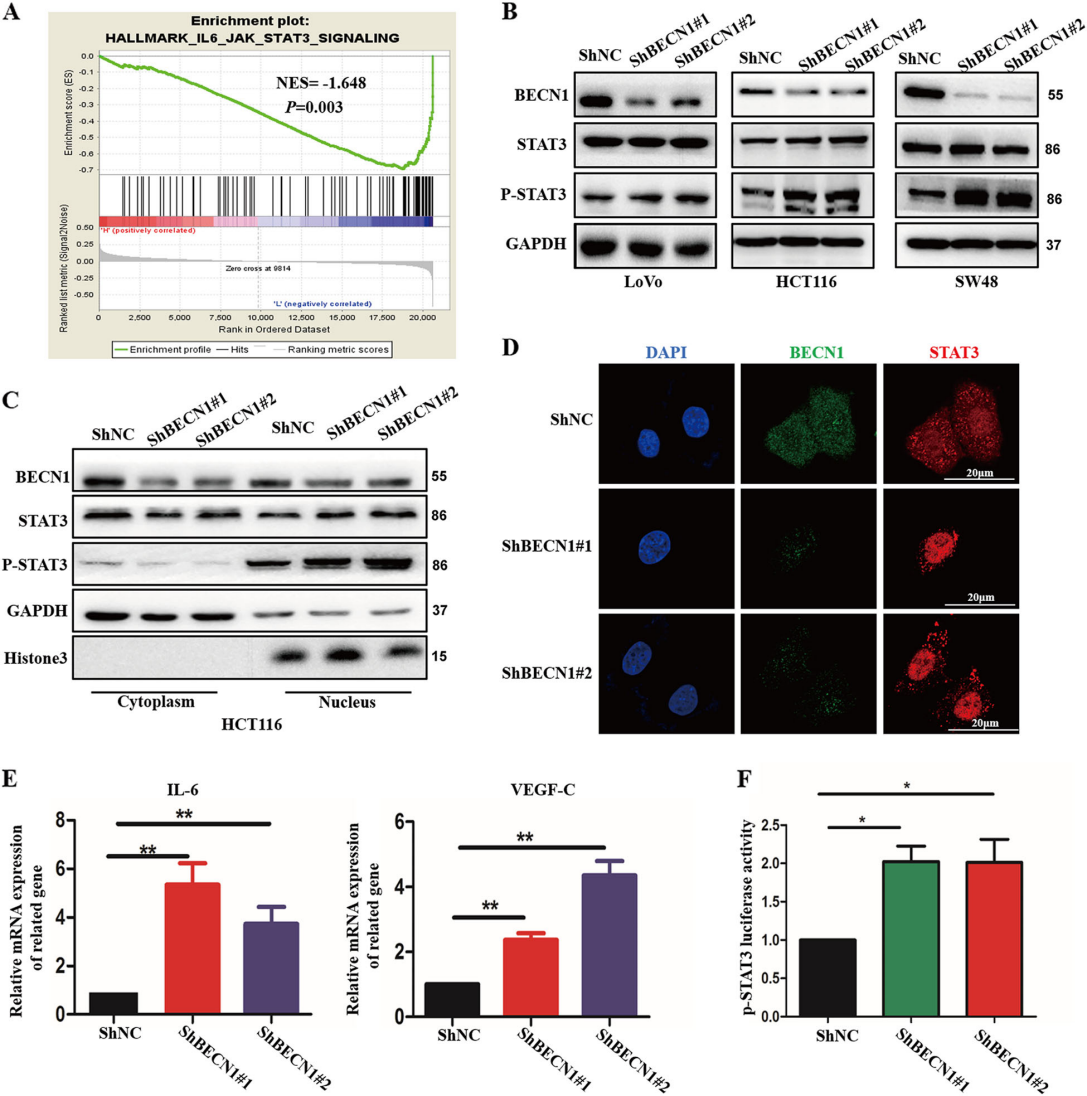
**Loss of BECN1 activates the phosphorylation of STAT3**. **a** GSEA plot indicating that BECN1 expression is inversely correlated with JAK2/STAT3 enrichment gene signatures in the GEO database (GSE17536). **b** Western blot analysis of the indicated proteins in LoVo, HCT116 and SW48 cells expressing shRNA-NC or shRNA-BECN1. **c** Western blot analysis was used to determine the level of nuclear STAT3 and p-STAT3 in HCT116 cells stably expressing negative control, shRNA-BECN1#1 or shRNA-BECN1#2. **d** An immunofluorescence assay was performed to examine STAT3 localization in HCT116 cells among the indicated groups. Scale bar: 20 μm. **e** qPCR was used to examine the expression of IL-6 and VEGF-C in the indicated HCT116 cells. **f** STAT3 luciferase activity was measured in the indicated HCT116 cells transfected with PGL6-p-STAT3 and pRL-TK plasmids after 24 h of incubation by a dual-luciferase assay. The values are the mean ± SD for triplicate samples (*P < 0.05 by an independent Student’s *t* test).

To further understand the potential molecular mechanism underlying the increased metastasis induced by decreased BECN1 expression, we surveyed previously reported potential signaling pathways involved in metastasis by gene set enrichment analysis (GSEA). By analyzing the previously published CRC patient microarray data, we interestingly found that the expression of BECN1 was inversely correlated with STAT3-activated gene signatures (Fig. 3a). To test the reliability of this outcome, we further examined several reference parameters, such as the normalized enrichment score (NES) and the *P* value shown in Fig. 3a. This prompted us to consider whether BECN1 regulates the STAT3 signaling pathway and then controls CRC progression. We found that knockdown of BECN1 markedly increased the phosphorylation levels of STAT3 in LoVo, HCT116, and SW48 cells (Fig. 3b). In addition, exogenous expression of BECN1 in SW48 cells significantly decreased the levels of STAT3 phosphorylation (Fig. S3A). As previously reported, STAT3 acts as a transcription factor, and phosphorylated STAT3 translocates into the nucleus to activate target genes. We examined whether loss of BECN1 expression might change the nuclear translocation of STAT3. As shown in Fig. 3c, knockdown of BECN1 significantly promoted the nuclear localization of both total and phosphorylated STAT3. Immunofluorescence (IF) results also showed that loss of BECN1 markedly increased the nuclear localization of STAT3 in HCT116 cells (Fig. 3d). In addition, the effect of BECN1 on STAT3 target genes was also determined. We showed that knockdown of BECN1 increased the STAT3-induced expression of IL-6 and VEGF-C, the canonical STAT3 signaling target genes (Fig. 3e). Furthermore, we employed a dual-luciferase assay and demonstrated that knockdown of BECN1 enhanced STAT3 transcriptional activity (Fig. 3f). Collectively, these data suggest that BECN1 might directly modulate STAT3 activity and regulate STAT3 nuclear localization in CRC.

### The effect of BECN1 on CRC metastasis depends on STAT3

**Fig. 4:**
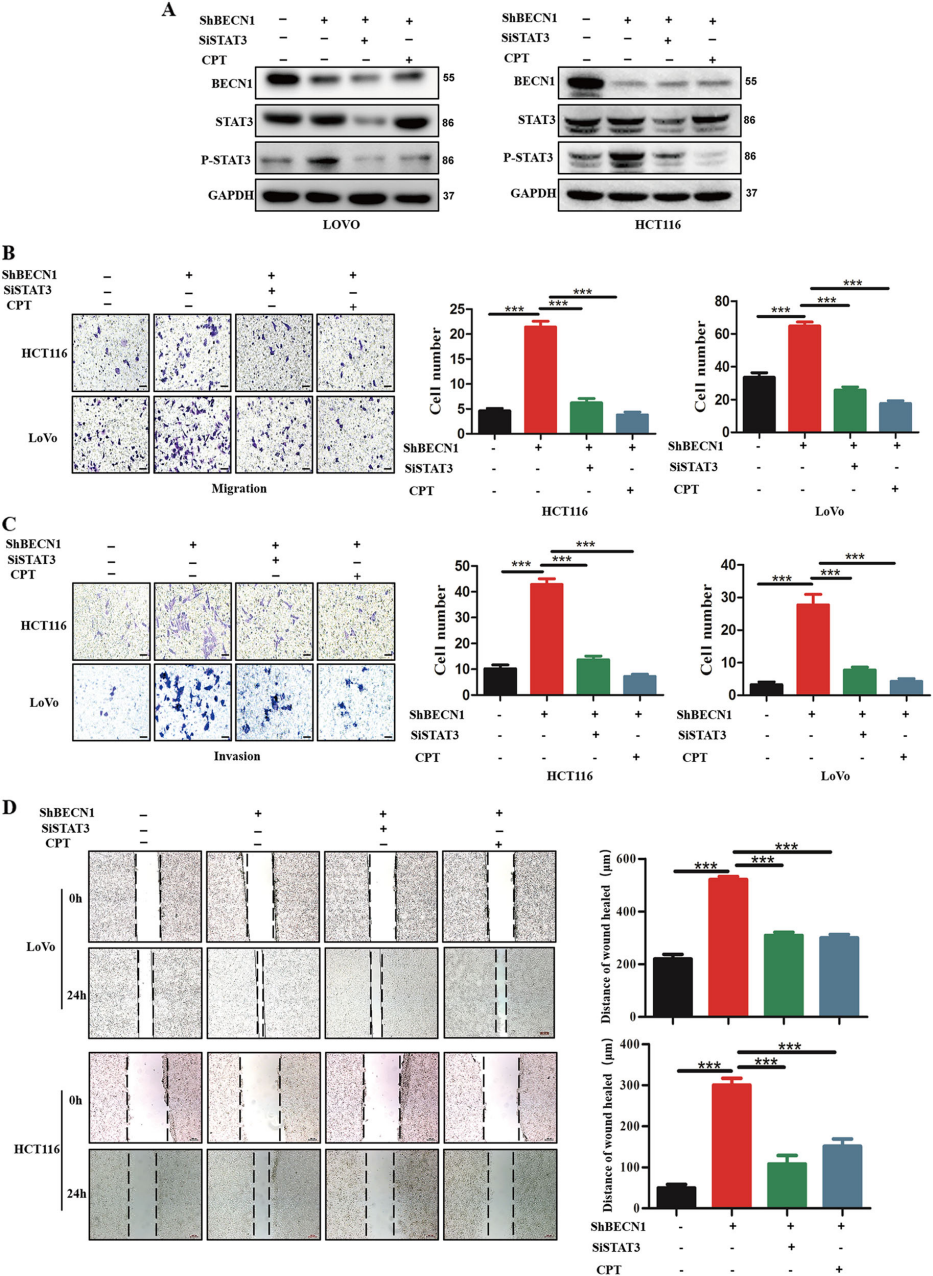
**The effect of BECN1 on CRC metastasis depends on STAT3**. **a** Western blotting assay showing the expression of proteins in the indicated cells. GAPDH was used as the loading control. **b**, **c** Cell migration and invasion assays in LoVo and HCT116 cells expressing negative control shRNA, shRNA-BECN1, as well as in those cells transfected with si-STAT3 or treated with CPT. The right panel shows the histograms of the results. The values are the mean ± SD for triplicate samples (*P < 0.05 by an independent Student’s *t* test). Scale bar: 100 μm. **d** Wound-healing assays in LoVo and HCT116 cells expressing control negative shRNA, shRNA-BECN1#1, or shRNA-BECN1#2, as well as in those transfected with si-STAT3 or shRNA-BECN1, after treatment with CPT. The right panel shows the histograms of the results. The values are the mean ± SD for triplicate samples (*P < 0.05 by an independent Student’s *t* test). Scale bar: 200 μm.

To explore the role of STAT3 in BECN1 signaling, we silenced endogenous STAT3 expression in both LoVo and HCT116 cells expressing shRNA-BECN1. We confirmed that the increased phosphorylation of STAT3 induced by knockdown of BECN1 was reversed by genetic or pharmacological inhibition of STAT3 (Fig. 4a). Importantly, we found that knockdown of BECN1 led to an increase in CRC cell migration and invasion; however, this effect could be reversed by the inhibition of STAT3 (Fig. 4b, c). In addition, pharmacological inhibition of STAT3 also reversed the enhanced migration and invasion induced by knockdown of BECN1 (Fig. 4b, c). The wound-healing assay also showed similar results (Fig. 4d).

Recent reports revealed that epithelial-to-mesenchymal (-like) transition (EMT) is involved in the metastasis mediated by STAT3 in CRC^22^. Interestingly, using GSEA, we found that the expression of BECN1 was inversely correlated with EMT-activated gene signatures (Fig. S3B), indicating that knockdown of BECN1 promotes CRC metastasis via regulation of the STAT3/EMT signaling pathway. To confirm this hypothesis, we further examined EMT-related protein expression. We showed that knockdown of BECN1 decreased the levels of E-cadherin (an epithelial marker) protein expression and increased the levels of vimentin (a mesenchymal marker) protein expression in HCT116 cells (Fig. S3C). These results further indicated that BECN1-mediated CRC metastasis was dependent on STAT3.

These data strongly demonstrated that BECN1-mediated CRC metastasis was dependent on the STAT3 signaling pathway.

### Knockdown of BECN1 decreases autophagic flux in CRC

**Fig. 5:**
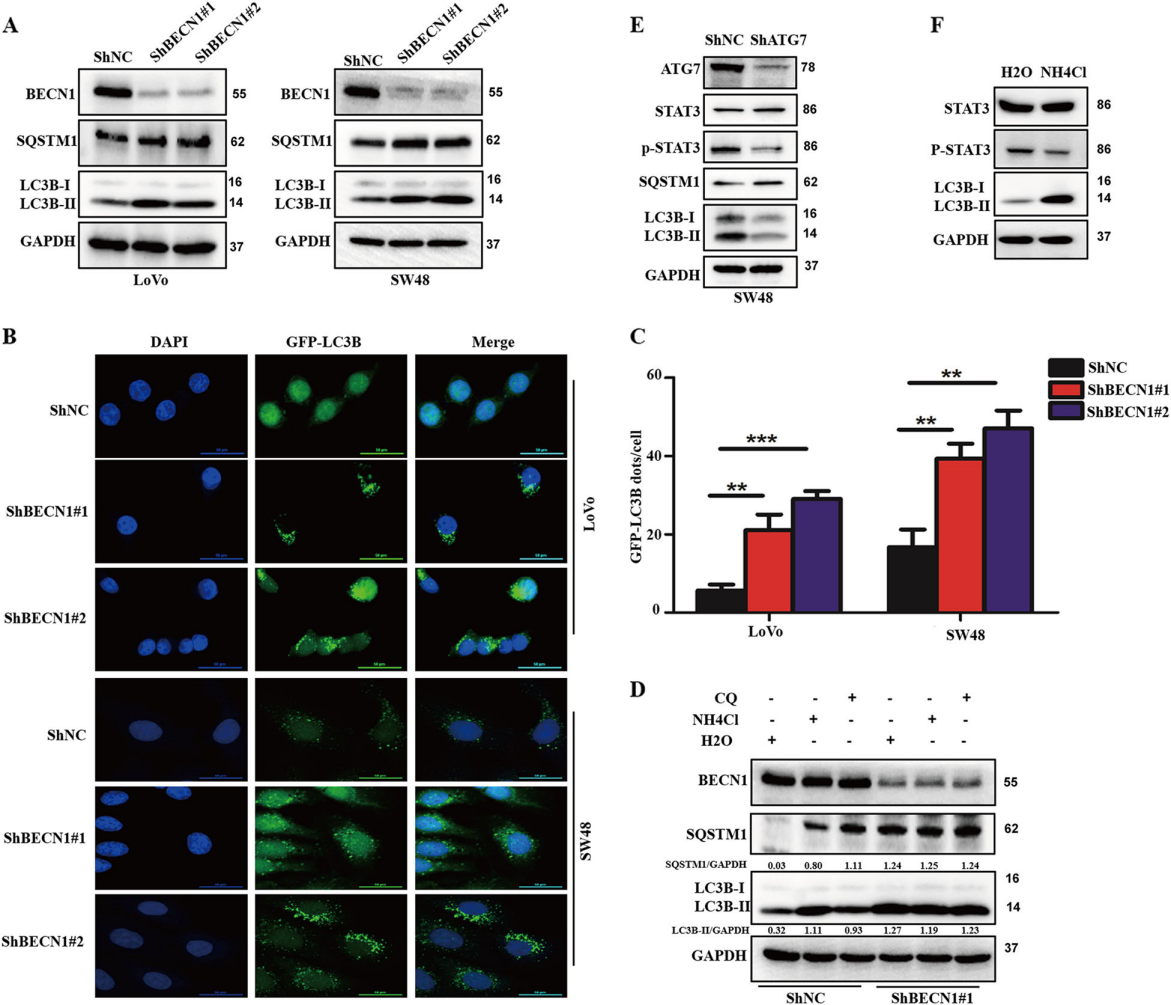
**Knockdown of BECN1 decreases autophagic flux in colorectal cancer**. **a** Western blot analysis used to determine the protein levels of LC3B, BECN1, SQSTM1/p62, and GAPDH in both LoVo and SW48 cells expressing the negative control RNA or shRNA-BECN1. **b**, **c** LoVo and SW48 cells expressing the negative control RNA or shRNA-BECN1 were transfected with GFP-LC3B plasmids for 48 h, and then the GFP-LC3B signal was examined under confocal microscopy. The amount of GFP-LC3B signal in each cell was quantified. Scale bar: 50 μm. **d** SW48 cells expressing the negative control RNA or shRNA-BECN1#1 were treated with or without 25 μM CQ for 2 h or 5 mM NH_4_Cl for 4 h. The accumulation of LC3B and SQSTM1was determined by a western blot assay and represented the autophagic flux. The LC3B accumulation and SQSTM1 was quantified using GAPDH as the loading control. The results indicate the mean ± SD for triplicate independent experiments (*P < 0.05, **P < 0.01, ***P < 0.001 by an independent Student’s *t* test). **e** SW48 cells were transfected with negative control siRNA (siNC) or siRNA-ATG7 (siATG7) for 72 h, and then cells were harvested for western blot assays. **f** SW48 cells were treated with H_2_O or 5 mM NH_4_Cl for 4 h, and then cells were harvested for western blot assays.

To explore the underlying mechanism and given the vital role of BECN1 in autophagy reported in mammals, we investigated the role of BECN1 in autophagy in CRC. As shown in Fig. 5a, knockdown of BECN1 increased the accumulation of LC3B-II (an marker of the autophagosome) and SQSTM1/p62 (a marker of autophagic flux) in both LoVo and SW48 cells, indicating that BECN1 might play an important role in autophagic flux in CRC. To further confirm this phenomenon, we transfected GFP-LC3B plasmids into cells and then examined the amount of GFP-LC3B dots to evaluate the levels of autophagosomes. Consistent with the western blot results, as shown in Fig. 5b, c, we clearly found that knockdown of BECN1 led to an increased amount of GFP-LC3B signal in CRC cells. Furthermore, to evaluate the effect of BECN1 on autophagy, we treated cells with chloroquine (CQ) and NH_4_Cl (both are autophagy inhibitors) to examine the levels of autophagic flux. Expectedly, we found that both autophagy inhibitors strongly elevated LC3B-II accumulation in the shNC groups but not in the sh-BECN1 groups, suggesting that BECN1 mainly plays a critical role in the later process of autophagosome maturation (autophagic flux) in CRC.

Many reports have shown that autophagic flux regulates the phosphorylation of STAT3, which prompted us to determine whether knockdown of BECN1 increased the phosphorylation of STAT3 by inhibiting autophagic flux. Therefore, we examined the effect of autophagic flux on the phosphorylation of STAT3. We found that both genetic inhibition of autophagic flux via silencing of ATG7, a critical gene regulating autophagic flux, and pharmacological inhibition of autophagic flux by NH_4_Cl decreased the phosphorylation of STAT3 (Fig. 5e, f), suggesting that autophagy promotes the phosphorylation of STAT3 in CRC. While knockdown of BECN1 decreased autophagic flux but promoted STAT3 phosphorylation in CRC, these findings indicated that BECN1 regulated STAT3 phosphorylation through an autophagy-independent mechanism.

### BECN1 interacts with STAT3 and attenuates the interaction between STAT3 and JAK2

**Fig. 6:**
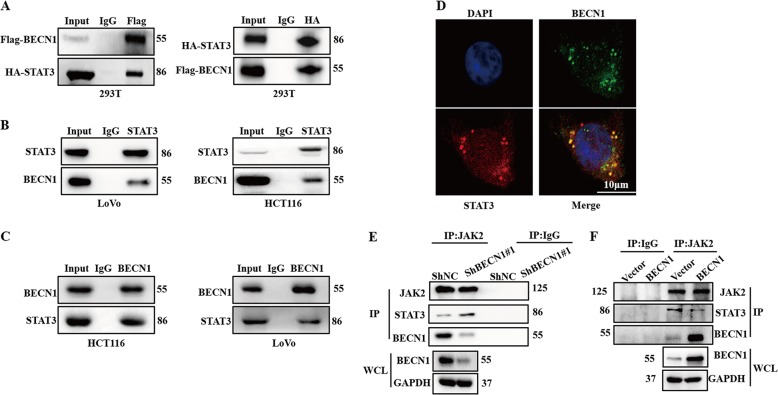
**BECN1 directly interacts with STAT3 and attenuates the interaction between STAT3 and JAK2**. **a** 293T cells were cotransfected with HA-STAT3 and FLAG-BECN1 plasmids, and immunoprecipitation experiments were used to examine the interaction between HA-STAT3 and FLAG-BECN1. **b**, **c** Immunoprecipitation was performed to determine the interaction between BECN1 and STAT3 in both HCT116 and LoVo cells. **d** Immunofluorescence staining showed the endogenous interaction between BECN1 and STAT3 in the cytoplasm of HCT116 cells. Scale bar: 20 μm. **e**, **f** Immunoprecipitation was performed with a JAK2 antibody, and western blot assays were used to examine the immunoprecipitated complexes, including JAK2, BECN1, and STAT3, among the indicated groups.

To further determine the mechanism by which knockdown of BECN1 increases the phosphorylation of STAT3, we cotransfected 293T cells with FLAG-BECN1 and HA-STAT3 plasmids and found that FLAG-BECN1 was coprecipitated with HA-STAT3 (Fig. 6a). In the transfection experiments, we also examined the interaction of endogenous BECN1 and STAT3. Similarly, the physical interaction between endogenous BECN1 and STAT3 was also observed in both LoVo and HCT116 cells (Fig. 6b, c). Further confirming this interaction, an IF assay also demonstrated that BECN1 mainly interacted with STAT3 in the cytoplasm in HCT116 cells (Fig. 6d).

STAT3 is known as a substrate of JAK2. Following phosphorylation in the cytoplasm, STAT3 translocates into the nucleus and targets gene expression^25^. Because the interaction between BECN1 and STAT3 occurs primarily in the cytoplasm, we hypothesized that BECN1 interferes with the interaction between STAT3 and JAK2. Next, we found that knockdown of BECN1 could enhance the binding of STAT3 to JAK2 in CRC cells (Fig. 6e) and that overexpression of BECN1 suppressed the interaction between STAT3 and JAK2 (Fig. 6f). Collectively, these data indicated that BECN1 directly bound to STAT3 and attenuated the interaction between STAT3 and JAK2, thus impacting the phosphorylation of STAT3.

### BECN1 downregulation promotes CRC metastasis in vivo

**Fig. 7:**
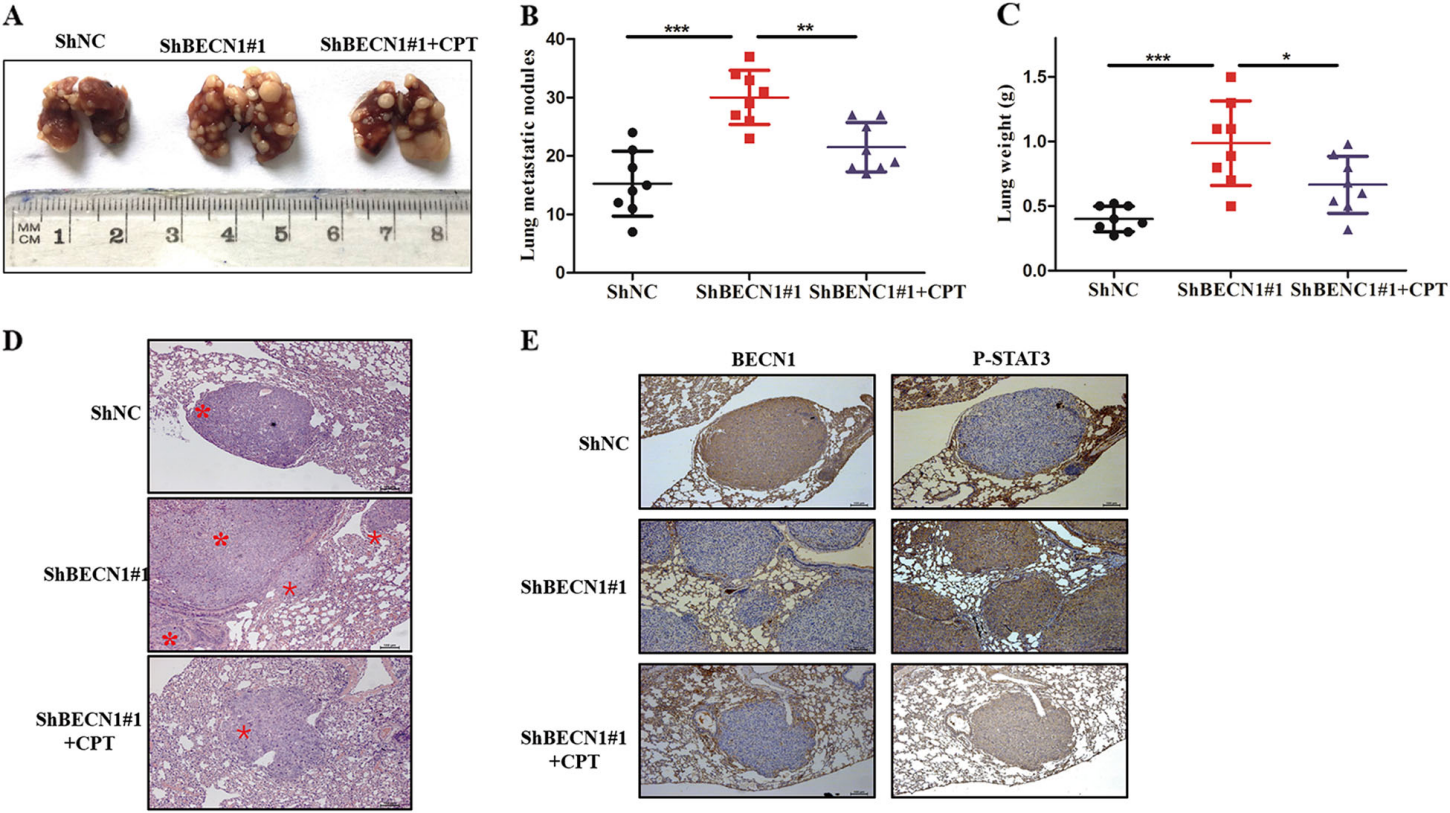
**BECN1 downregulation promotes CRC metastasis in vivo**. **a** Nude mice were intravenously injected via the tail vein with HCT116 cells expressing negative control negative ShRNA, ShRNA-BECN1#1, and ShRNA-BECN1#1, as well as tail-vein injection with the special inhibitor of STAT3 (CPT). Representative images of lungs showing metastatic foci generated from HCT116 cells and (**b**, **c**) quantitative analysis of the average number of metastatic foci and the wet weight of lungs in three groups. **d**, **e** Representative HE and representative IHC images of BECN1 and p-STAT3 expression in HCT116 cell lung metastatic foci. Scale bar: 100 μm.

In an effort to investigate the effect of BECN1 on metastasis in vivo, HCT116 control and HCT116 sh-BECN1 cells were intravenously injected into nude mice. At 8 weeks after injection, all nude mice were sacrificed, and the lungs were harvested. We found that the weight differences among groups was not significant (data not shown). However, the number of metastatic nodules formed by HCT116 sh-BECN1 cells was much higher than that for the control group (Fig. 7a, c). Furthermore, this significant change was reversed by pharmacological inhibition of STAT3 (Fig. 7a, c). IHC staining showed similar results (Fig. 7d). We also determined the wet lung weight between the two groups; the data showed that the wet lung weight of the sh-BECN1 group was heavier than that of the control group (Fig. 7c). We examined the association between p-STAT3 and BECN1 protein expression in lung metastatic nodules by IHC staining, and the data indicated that the expression of p-STAT3 was enhanced with knockdown of BECN1 in vivo (Fig. 7d). Taken together, the above results suggested that knockdown of BECN1 enhanced metastasis in a manner dependent on the STAT3 signaling pathway.

## Discussion

Beclin1 is an autophagic protein that plays a critical role in the formation of autophagosomes^26,27^. Increasing evidence has shown that BECN1 is involved in many signaling pathways in addition to its involvement in the regulation of autophagy, and an increasing number of reports have shown that BECN1 is associated with poor prognosis and metastasis^28,29^. Here, we also showed that the expression of BECN1 was lower in CRC samples than in adjacent normal tissues and was associated with poor prognosis in CRC, indicating that BECN1 could act as a suppressor and a novel prognostic marker for colon cancer patients. Our data indicate that knockdown of BECN1 has no influence on cancer cell growth but markedly increases mobility and invasion. Interestingly, the role of BECN1 in CRC samples has varied across studies, which may be related to the different experimental techniques used in different laboratories as well as the different efficiencies of accurately measuring low expression of BECN1^30,31^. This finding also suggests that BECN1 may have opposite effects at different stages of CRC progression.

STAT3 is also abnormally activated in many kinds of cancers^32,33^. The JAK2/STAT3 signaling pathway has been widely studied in different steps of tumor development and may act as a promising molecular therapeutic target^34,35^. Matjaz R. suggested that the IL-6R/STAT3/miR-34a feedback loop promotes colon cancer metastasis by inducing EMT. Here, we showed that knockdown of BECN1 increased the phosphorylation of STAT3 and promoted its nuclear import. Interestingly, a functional link between BECN1 knockdown and STAT3 inactivation was suggested in transformed T lymphocytes^36^, which seems to be contrary to our data. We thought that this discrepancy might be due to cellular genetic backgrounds. It is worth noting that increasing evidence has shown that the role of BECN1 in tumor development varies depending on the cellular context^37^. Meanwhile, many signaling pathways have also shown different effects in different kinds of tumor^38^. Thus, it was reasonable that knockdown of BECN1 increased the phosphorylation of STAT3 in CRC. Furthermore, functional experiments demonstrated that the effect of knockdown BECN1 on cancer cells was reversed by genetic or pharmacological inhibition of STAT3, strongly indicating that BECN1 is involved in CRC metastasis through regulation of STAT3 phosphorylation in CRC. Notably, numerous reports have demonstrated that STAT3 is involved not only in tumor metastasis but also in proliferation, the cell cycle and apoptosis^39–41^. Herein, we found that BECN1 influenced the CRC metastasis mediated by STAT3. However, BECN1 regulated STAT3 phosphorylation in different CRC cell lines. BECN1 had no effect on CRC proliferation, the cell cycle or apoptosis. These results indicate that BECN1 might regulate other signaling pathways that are also involved in proliferation, the cell cycle and apoptosis in CRC and counteract the effect of the STAT3 signaling pathway on proliferation, the cell cycle, and apoptosis in CRC.

Emerging evidence has shown that autophagy can influence STAT3 phosphorylation. For example, Maycotte showed that in MCF7 cells, autophagy promoted the phosphorylation of STAT3. However, in MDA-MB-468 cells, autophagy inhibited the phosphorylation of STAT3^42^, suggesting that autophagy regulates the phosphorylation of STAT3 in a cell-type-dependent manner. BECN1 acts as an autophagy protein and plays multiple roles in autophagy. However, the role of BECN1 in autophagy and the effect of autophagy on the phosphorylation of STAT3 in CRC are still unclear. Here, we illustrated that knockdown of BECN1 decreased the autophagic flux in CRC and that inhibition of autophagy attenuated the levels of STAT3 phosphorylation, suggesting that BECN1 regulates STAT3 phosphorylation independently of autophagy in CRC and that the role of BECN1 in CRC metastasis might depend on autophagy. To further determine the precise mechanism of regulation of CRC metastasis by BECN1, we found that both BECN1 and STAT3 bound to JAK2 and that BECN1 disrupted the interaction between STAT3 and JAK2, thereby influencing the phosphorylation of STAT3 in CRC. In vivo experiments confirmed that BECN1 downregulation promoted CRC metastasis. Importantly, CPT (a STAT3 inhibitor) markedly decreased the lung metastatic nodules induced by knockdown of BECN1, suggesting that pharmacological inhibition of STAT3 is a promising method for advanced CRC patients with metastasis. These data strongly suggest that the JAK2/STAT3 signaling pathway is required for the CRC metastasis mediated by knockdown of BECN1. These findings also ruled out the hypothetical explanation that BECN1 plays distinct roles in multiple cellular processes beyond autophagy through involvement in different signaling pathways.

Taken together, our data provide evidence that knockdown of BECN1 triggers metastasis in CRC and that this function is mediated by upregulation of the phosphorylation of STAT3 and abrogation of the interaction between STAT3 and JAK2. In addition, the increased phosphorylation of STAT3 induced by knockdown of BECN1 was independent of autophagy. Furthermore, pharmacological inhibition of STAT3 attenuated the CRC metastasis mediated by silencing of BECN1 in vivo. These results suggest that the novel signaling pathway BECN1/JAK2/STAT3 may be a potentially effective therapeutic target for advanced CRC patients with metastasis.

## Materials and methods

### Reagents and antibodies

CQ (Sigma, C6628) was dissolved in phosphate buffered saline (PBS, Sigma, 08057), and NH_4_Cl (Sigma, 09718) was dissolved in sterile H_2_O. Cryptotanshinone (CPT) was obtained from MedChemExpress (MCE, HY-N0174). ATG7, GAPDH, STAT3, and JAK2 were obtained from Santa Cruz Biotechnology (sc-376212, sc-47724, sc-8019, and sc-390539). E-cadherin, vimentin, BECN1, STAT3, and p-STAT3 were purchased from Cell Signaling Technology (3195, 5741, 3738, 4904, and 9145).

### Cell culture

HCT116, LoVo, SW48, and 293T embryonic kidney cells were purchased from the American Type Culture Collection. All cells were cultured in Dulbecco’s modified Eagle’s medium (DMEM, containing 4.5 g/l of glucose; Gibco, 11995–065) supplemented with 10% fetal bovine serum (FBS, Gibco, 16140–071), 100 units/ml of penicillin, and 100 μg/ml of streptomycin (Invitrogen, 15140148) with 5% CO_2_ at 37 °C. HCT116, LoVo, and SW48 cells were transduced with lentiviruses expressing shRNA-BECN1 or control shRNA and then selected with 2 μg/ml puromycin for 2 days. The shRNA-BECN1 target sequences were as follows: shRNA-BECN1#1: 5′-CCGGCCCGTGGAATGGAATGAGATTCTCGAGAATCTCATTCCATTCCACGGGTTTTTG-3′; and shRNA-BECN1#2: 5′-CCGGCTCAAGTTCATGCTGACGAATCTCGAGATTCGTCAGCATGAACTTGAGTTTTTG-3′.

### Cell viability assay

A total of 5 × 10^3^ cells were plated in 96-well plates. At the indicated time points, 10 μl of CCK8 reagent (MCE, HY-K0301) was added to the cells according to the manufacturer’s instructions. After incubation for 2 h at 37 °C, the cell growth was measured at 450 nm.

### Flow cytometric analysis

Cells were plated into 6-well plates and cultured to 90% confluence. Then, the cells were harvested and stained with an annexin V-FITC/PI staining kit according to the manufacturer’s instructions (Sigma-Aldrich). After 0.5 h, the treated cells were analyzed by flow cytometry to examine apoptosis (BD Biosciences). For cell cycle analysis, cells were plated into 6-well plates and cultured to 90% confluence. Then, the cells were fixed with iced 80% ethanol overnight. Next, cells were incubated with RNase and PI. After 0.5 h, the cells were analyzed by flow cytometry to examine apoptosis (BD Biosciences). These experiments were repeated three times independently.

### Tissue samples and ethical approval

The tissue samples used in this study were obtained from Tongji Hospital, and patients were clinically and histopathologically diagnosed at Tongji Hospital. Written informed consent was obtained from individuals prior to this experiment and approved by the research medical ethics committee of Huazhong University of Science and Technology.

### RNA isolation and quantitative real-time PCR (qRT-PCR)

Briefly, total RNA was extracted with TRIzol reagent (Invitrogen, 15596–026) according to the manufacturer’s protocol. The first cDNA was synthesized with PrimeScript^™^ RT Master Mix (Takara, RR036A). Then, qRT-PCR was performed with SYBR Green SuperMix (TransGen Biotech, AQ101-02). Relative mRNA expression levels were normalized to GAPDH with the 2^−ΔΔCT^ method. The qRT-PCR specific primers used here are as follows: upstream primer 5′-ACTCACCTCTTCAGAACGAATTG-3′ and downstream primer 5′-CCATCTTTGGAAGGTTCAGGTTG-3′ for the IL-6 gene; upstream primer 5′-ATGTGTGTCCGTCTACAGATGT-3′ and downstream primer 5′-GGAAGTGTGATTGGCAAAACTGA-3′ for the VEGFC gene; and upstream primer 5′-ACAACTTTGGTATCGTGGAAGG-3′ and downstream primer 5′-GCCATCACGCCACAGTTTC-3′ for the GAPDH gene.

### The luciferase reporter assay

A total of 2 × 10^5^ cells were seeded into 12-well plates and transfected with a STAT3-reporter luciferase plasmid. After transfection for 48 h, the luciferase assays were measured using a Dual-Luciferase Assay Kit (Promega, 16181). Three independent experiments were performed.

### Western blotting and coimmunoprecipitation (co-IP)

The cells were harvested at the indicated times and lysed in RIPA cell lysis buffer at 4 °C. Then, the cell lysates were centrifuged for 15 min at 120,00 × *g* at 4 °C, and the protein in the supernatant was quantified by a bicinchoninic acid assay kit (Pierce, 23225). Briefly, equal amounts of protein were loaded and separated on a 10 or 12% sodium dodecyl sulfate-polyacrylamide gradient gel. After being transferred onto polyvinylidene fluoride (PVDF) membranes (Millipore, K5KA5020C) and blocked with 5% nonfat milk for 1 h, the membranes were incubated with primary antibodies and corresponding horseradish peroxidase-conjugated secondary antibodies. Finally, the bands were visualized using a SuperSignal West Femto Substrate Trial Kit (Thermo Scientific, 34096). For the co-IP experiment, briefly, the cell supernatants were pretreated with protein A/G agarose (Santa Cruz Biotechnology, sc-2003) beads for 1 h at 4 °C. Then, 2 μg of the corresponding primary antibody was added to the supernatants overnight at 4 °C. Next, 50 μl of protein A/G agarose was added for 4 h. The beads were washed more than three times using ice-cold PBS or cell lysis buffer, and the bound proteins were boiled in loading buffer for further analysis.

### Cellular fractionation

Cellular fractions were extracted by a nuclear protein and cytoplasmic protein extraction kit. In brief, cells were collected and suspended in buffer A (Beyotime Biotechnology, P0027), aggressively vibrated for 15 min on ice, and centrifuged at 12,000 × *g* for 5 min. The supernatant, representing the cytoplasmic fraction, was collected. The residual cell pellet was washed twice, then fully resuspended in buffer B (Beyotime Biotechnology, P0027) and aggressively vibrated for 10 min at 4 °C. The supernatant, representing the nuclear extract, was collected.

### Wound-healing assay

Cells were plated into six-well plates overnight and were carefully scratched with sterile 200 μl pipette tips. Cells were observed every 24 h using a microscope. For the transwell migration assay, approximately 40,000 cells suspended in 200 μl of serum-free medium were seeded into the upper chamber, and complete medium supplemented with 10% FBS was added into the lower chamber. After incubation for 16 h, the cells on the lower surface were fixed with 4% methanol, stained with 0.1% crystal violet and counted using a microscope. At least three visual areas were photographed.

### Migration and invasion assays

Briefly, cells (2 × 10^4^ for the migration assay and 4 × 10^4^ for the invasion assay) in 200 μl of serum-free medium were seeded into the top chamber of a noncoated membrane or Matrigel-coated transwell chambers (BD Biosciences, 6140322). Medium supplemented with 20% FBS was added to the lower chamber. After incubation for 24 h, the cells on the lower surface of the membrane were fixed with methanol for 15 min and stained with 0.1% crystal violet for 15 min at room temperature. The cells were observed using a microscope.

### RNA interference

A negative control small interfering RNA (siRNA) and siRNA-stat3 were obtained from RIBo. Transfection was performed according to the manufacturer’s protocol. The target sequence used in this study was as follows: 5′-GCACAATCTACGAAGAATCAA-3′.

### Immunofluorescence assay

A total of 5 × 10^5^ cells were seeded into 24-well plates with a preplaced glass slide. After 24 h, cells were transfected with GFP-LC3B plasmids. Then, the cells were fixed with methanol for 15 min at room temperature and washed twice with PBS. Next, the cells were stained with 4,6-diamidino-2-phenylindole (DAPI) to visualize the nuclei and washed twice with PBS. Finally, cells were detected under a Zeiss LSM 510 laser confocal microscope (Zeiss, Oberkochen, Baden-Württemberg, Germany). The GFP-LC3B signal was analyzed by ImageJ software.

### Animal experiments

Four-week-old male nude mice were purchased from HuaFukang Bioscience Company (Beijing, China) and were intravenously injected with HCT116 cells (2 × 10^6^) stably expressing negative control shRNA or shRNA-BECN1#1. Every other day, one group was intravenously injected with CPT in a volume of 10 ml/kg body weight; the others were intravenously injected with an equal volume of PBS. The mice were weighed once every week. After 8 weeks, mice were sacrificed, and wet lungs were harvested for weighing and analysis. All animal experiments were approved by the Institutional Animal Care and Use Committee of Tongji Hospital, Huazhong University of Science and Technology.

### Statistical analysis

GraphPad Prism 5.0 (GraphPad Software, Inc., La Jolla, CA, USA) was used for statistical analysis. The results are shown as the means ± standard deviations (SDs). Each experiment was performed at least three times. *P* values of <0.05 were considered significant.

## Supplementary information

Supplementary Figure Legends

Supplementary Figure 1

Supplementary Figure 2

Supplementary Figure 3

## References

[1] Liang XH (1999). Induction of autophagy and inhibition of tumorigenesis by beclin 1. Nature.

[2] Qian X (2017). Phosphoglycerate Kinase 1 Phosphorylates Beclin1 to Induce Autophagy. Mol. Cell.

[3] Stjepanovic G, Baskaran S, Lin MG, Hurley JH (2017). Vps34 kinase domain dynamics regulate the autophagic PI 3-kinase complex. Mol. Cell.

[4] Matsunaga K (2009). Two Beclin 1-binding proteins, Atg14L and Rubicon, reciprocally regulate autophagy at different stages. Nat. Cell Biol..

[5] Liang XH (1998). Protection against fatal Sindbis virus encephalitis by beclin, a novel Bcl-2-interacting protein. J. Virol..

[6] Oberstein A, Jeffrey PD, Shi Y (2007). Crystal structure of the Bcl-XL-Beclin 1 peptide complex: Beclin 1 is a novel BH3-only protein. J. Biol. Chem..

[7] Erlich S (2007). Differential interactions between Beclin 1 and Bcl-2 family members. Autophagy.

[8] Hurley JH, Schulman BA (2014). Atomistic autophagy: the structures of cellular self-digestion. Cell.

[9] Levine B, Kroemer G (2008). Autophagy in the pathogenesis of disease. Cell.

[10] Hamacher-Brady A (2012). Autophagy regulation and integration with cell signaling. Antioxid. Redox Signal.

[11] Degenhardt K (2006). Autophagy promotes tumor cell survival and restricts necrosis, inflammation, and tumorigenesis. Cancer Cell.

[12] Huang T (2017). MST4 phosphorylation of ATG4B regulates autophagic activity, tumorigenicity, and radioresistance in glioblastoma. Cancer Cell.

[13] Cicchini M (2014). Autophagy regulator BECN1 suppresses mammary tumorigenesis driven by WNT1 activation and following parity. Autophagy.

[14] Gong, C. et al. Beclin 1 and autophagy are required for the tumorigenicity of breast cancer stem-like/progenitor cells. *Oncogene* **32**, 2261–2272, 2272e.2261-2211 (2013). 10.1038/onc.2012.252 PMC3679409 22733132

[15] Qu X (2003). Promotion of tumorigenesis by heterozygous disruption of the beclin 1 autophagy gene. J. Clin. Invest..

[16] Zhou WH (2012). Low expression of Beclin 1, associated with high Bcl-xL, predicts a malignant phenotype and poor prognosis of gastric cancer. Autophagy.

[17] Nicotra G (2010). Autophagy-active beclin-1 correlates with favourable clinical outcome in non-Hodgkin lymphomas. Mod. Pathol..

[18] Catlett-Falcone R, Dalton WS, Jove R (1999). STAT proteins as novel targets for cancer therapy. Signal transducer an activator of transcription. Curr. Opin. Oncol..

[19] Schindler C, Darnell JE (1995). Transcriptional responses to polypeptide ligands: the JAK-STAT pathway. Annu. Rev. Biochem..

[20] Bromberg JF (1999). Stat3 as an oncogene. Cell.

[21] Yang J (2005). Novel roles of unphosphorylated STAT3 in oncogenesis and transcriptional regulation. Cancer Res..

[22] Singh M (2015). STAT3 pathway regulates lung-derived brain metastasis initiating cell capacity through miR-21 activation. Oncotarget.

[23] Rokavec M (2014). IL-6R/STAT3/miR-34a feedback loop promotes EMT-mediated colorectal cancer invasion and metastasis. J. Clin. Invest..

[24] Yin Y (2017). The Immune-microenvironment confers chemoresistance of colorectal cancer through macrophage-derived IL6. Clin. Cancer Res..

[25] Yi W (1996). Growth hormone receptor cytoplasmic domain differentially promotes tyrosine phosphorylation of signal transducers and activators of transcription 5b and 3 by activated JAK2 kinase. Mol. Endocrinol..

[26] Hill SM, Wrobel L, Rubinsztein DC (2019). Post-translational modifications of Beclin 1 provide multiple strategies for autophagy regulation. Cell Death Differ..

[27] Menon MB, Dhamija S (2018). Beclin 1 Phosphorylation - at the Center of Autophagy Regulation. Front. Cell Dev. Biol..

[28] Wu S (2015). Expression and clinical significances of Beclin1, LC3 and mTOR in colorectal cancer. Int J. Clin. Exp. Pathol..

[29] Zhang MY (2014). Beclin 1 expression is closely linked to colorectal carcinogenesis and distant metastasis of colorectal carcinoma. Int. J. Mol. Sci..

[30] Shen H (2018). Knockdown of Beclin-1 impairs epithelial-mesenchymal transition of colon cancer cells. J. Cell Biochem..

[31] Zhang MY (2019). Effects of Beclin 1 overexpression on aggressive phenotypes of colon cancer cells. Oncol. Lett..

[32] Chang Q (2013). The IL-6/JAK/Stat3 feed-forward loop drives tumorigenesis and metastasis. Neoplasia.

[33] Marotta LL (2011). The JAK2/STAT3 signaling pathway is required for growth of CD44(+)CD24(-) stem cell-like breast cancer cells in human tumors. J. Clin. Invest..

[34] Schroeder A (2014). Loss of androgen receptor expression promotes a stem-like cell phenotype in prostate cancer through STAT3 signaling. Cancer Res..

[35] Yu H, Lee H, Herrmann A, Buettner R, Jove R (2014). Revisiting STAT3 signalling in cancer: new and unexpected biological functions. Nat. Rev. Cancer.

[36] Chen L, Liu D, Zhang Y, Zhang H, Cheng H (2015). The autophagy molecule Beclin 1 maintains persistent activity of NF-kappaB and Stat3 in HTLV-1-transformed T lymphocytes. Biochem. Biophys. Res. Commun..

[37] Gong C, Song E, Codogno P, Mehrpour M (2012). The roles of BECN1 and autophagy in cancer are context dependent. Autophagy.

[38] Roy N (2015). Brg1 promotes both tumor-suppressive and oncogenic activities at distinct stages of pancreatic cancer formation. Genes Dev..

[39] Su W (2019). LncRNA MIR22HG abrogation inhibits proliferation and induces apoptosis in esophageal adenocarcinoma cells via activation of the STAT3/c-Myc/FAK signaling. Aging.

[40] Xue X (2016). Iron uptake via DMT1 integrates cell cycle with JAK-STAT3 signaling to promote colorectal tumorigenesis. Cell Metab..

[41] Bollrath J (2009). gp130-mediated Stat3 activation in enterocytes regulates cell survival and cell-cycle progression during colitis-associated tumorigenesis. Cancer Cell.

[42] Maycotte P (2014). STAT3-mediated autophagy dependence identifies subtypes of breast cancer where autophagy inhibition can be efficacious. Cancer Res..

