# Supplementary material for: The autophagy-independent role of BECN1 in colorectal cancer metastasis through regulating STAT3 signaling pathway activation
Source: Cell Death Dis. 2020 May 1;11(5):304. doi: 10.1038/s41419-020-2467-3 (PMC7195408; doi:10.1038/s41419-020-2467-3)
Supplement: Supplementary file 1 — Supplementary Figure Legends [file 41419_2020_2467_MOESM1_ESM.docx]

**Legends**

**Figure1. BECN1 was downregulated in CRC.**

(A) TCGA colorectal cancer RNAseq database showing the expression of BECN1 in colorectal cancer and normal tissues. (B) Representative IHC pictures of BECN1 expression in CRC tumor and normal from the human protein atlas ([www.proteinatlas.org](http://www.proteinatlas.org/)).

**Figure2. BECN1 had no effect on cell cycle and apoptosis in CRC**

(A) Flow cytometric analysis showed that knockdown of BECN1 had no effect on cell cycle in LoVo and HCT116 cells. FigureS2: (A) Flow cytometric analysis showed that knockdown of BECN1 had no effect on apoptosis in LoVo and HCT116 cells.

**Figure3. BECN1 regulated EMT via modulation the phosphorylation of STAT3 in CRC.**

(A) Western blot assay showed that overexpression of BECN1 decreased the phosphorylation of STAT3 in SW48 cells. (B) Gene set enrichment analysis showed that the expression of BECN1 was inversely correlated with the EMT-activated gene signatures in GEO database (GSE17536). (C) Western blot analysis was used to determine the levels of Vimentin and E-cadherin in HCT116 cells stably expressing negative control, ShRNA-BECN1#1 and ShRNA-BECN1#2.
