# Supplementary figures and images for: The autophagy-independent role of BECN1 in colorectal cancer metastasis through regulating STAT3 signaling pathway activation
Source: Cell Death Dis. 2020 May 1;11(5):304. doi: 10.1038/s41419-020-2467-3 (PMC7195408; doi:10.1038/s41419-020-2467-3)

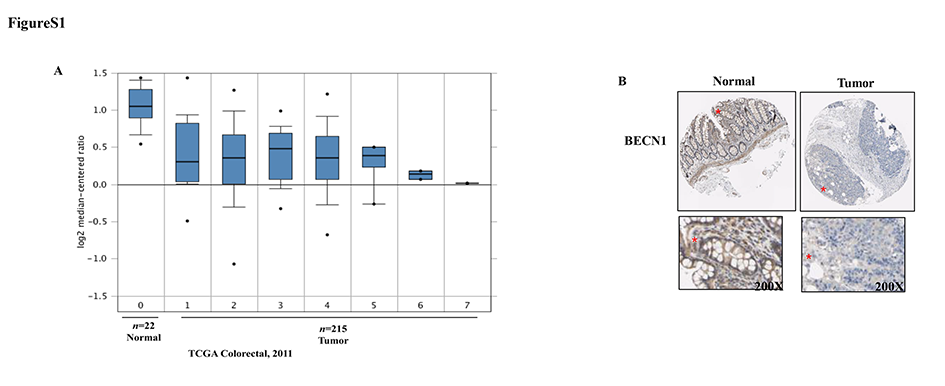

Supplement: Supplementary file 2 — Supplementary Figure 1 [file 41419_2020_2467_MOESM2_ESM.tif]

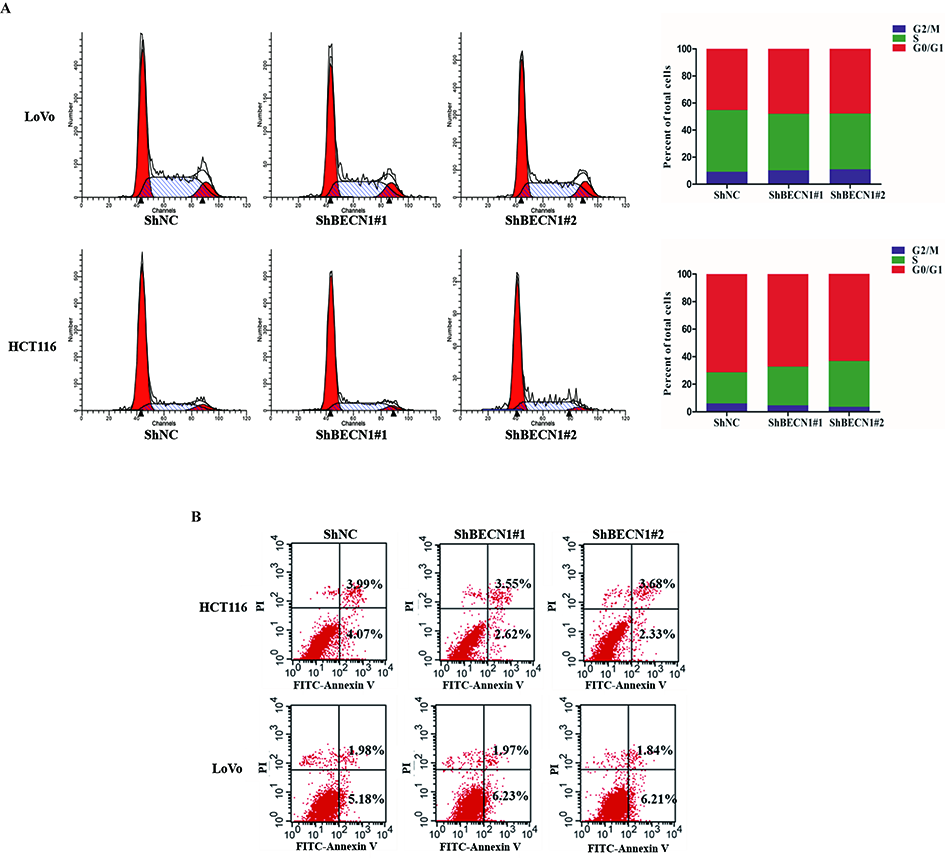

Supplement: Supplementary file 3 — Supplementary Figure 2 [file 41419_2020_2467_MOESM3_ESM.tif]

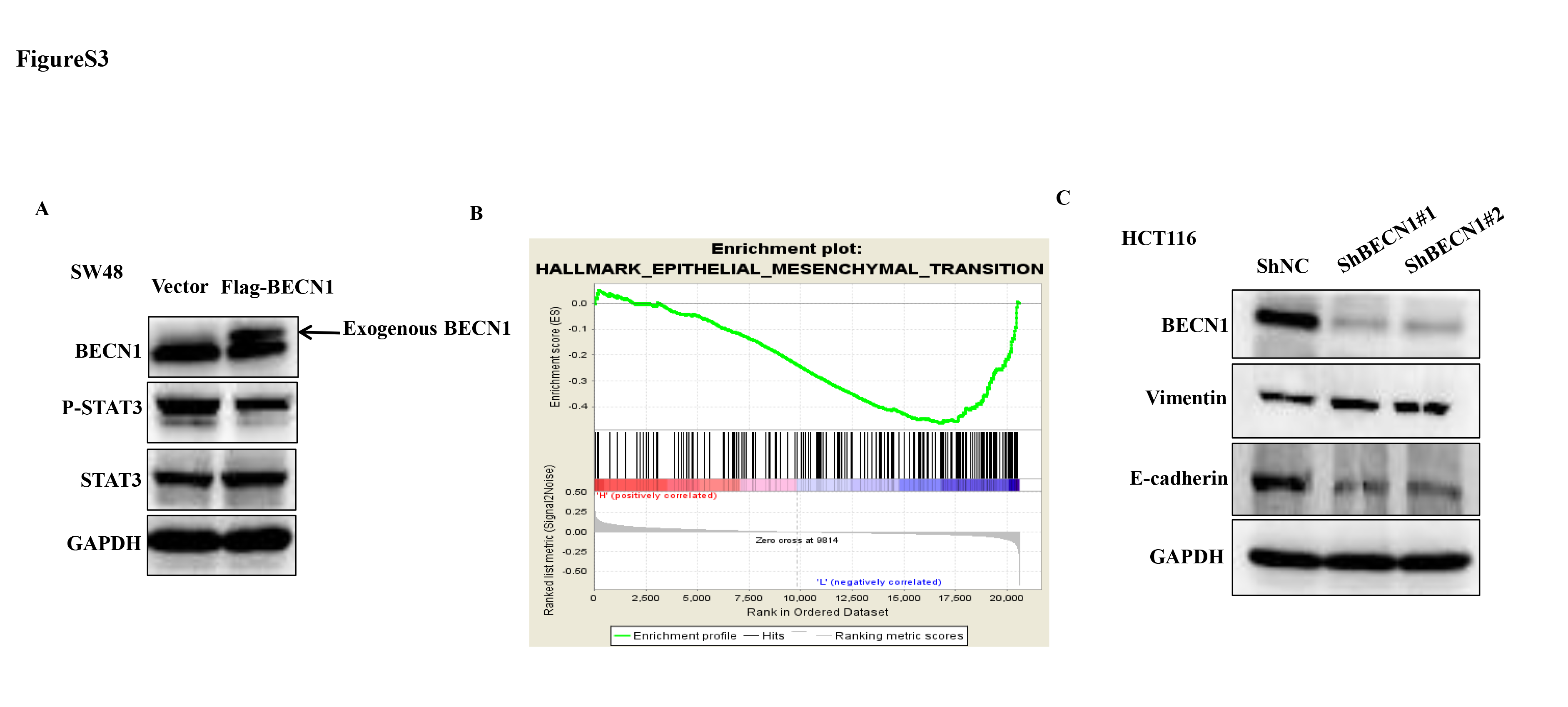

Supplement: Supplementary file 4 — Supplementary Figure 3 [file 41419_2020_2467_MOESM4_ESM.tif]
